# Supplementary material for: Fast objective coupled planar illumination microscopy
Source: Nat Commun. 2019 Oct 2;10:4483. doi: 10.1038/s41467-019-12340-0 (PMC6775063; doi:10.1038/s41467-019-12340-0)
Supplement: Supplementary file 1 — Supplementary Information [file 41467_2019_12340_MOESM1_ESM.pdf]

Supplementary information for
Fast Objective Coupled Planar Illumination Microscopy

Cody J. Greer and Timothy E. Holy

Supplementary Note 1: Aberrations for non-focal imaging

We analyze the effect on image quality when imaging outside of the native focal plane of an imaging system – referred to here as non-focal imaging. We begin by considering a conventional microscope imaging system configured to acquire an image of the native focal plane of the objective (diagrammed below, top). Modern microscopes are usually implemented with two lenses: an objective lens that faces the sample, and a tube lens that focuses light collected by the objective onto a camera sensor (or alternatively an eyepiece). The objective lens is designed to achieve diffraction-limited imaging of a single (native) object plane. Likewise a conventional tube lens forms a well-resolved image at a single image plane. Images of non-native object planes are also formed at non-native image planes (diagrammed in magenta) but in a conventional microscope these planes are not imaged because the camera sensor is coincident with the native plane.

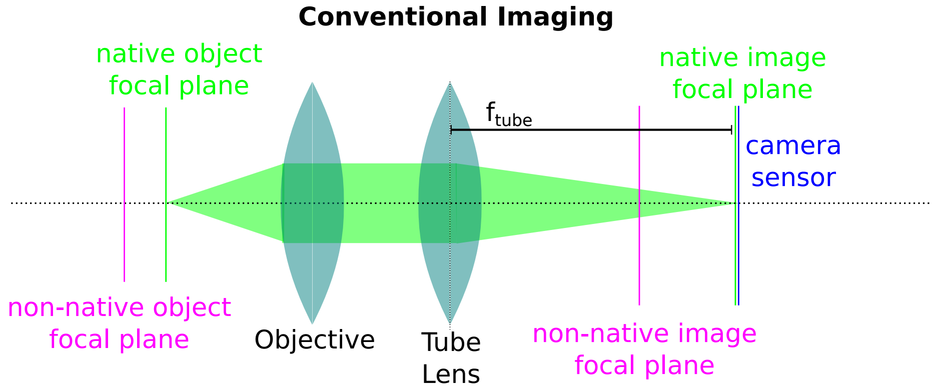

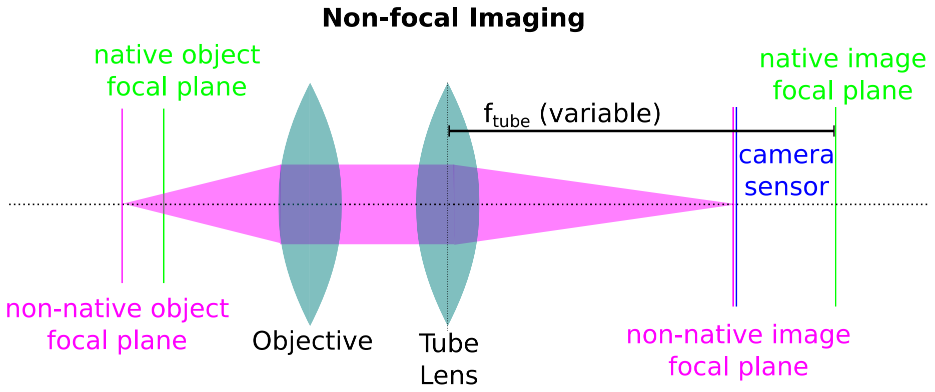

Some recently-developed microscopes bring these non-native planes into focus at the camera sensor by using a tube lens with a tuneable focal length (diagrammed below, bottom). Modern tuneable lenses can be adjusted very quickly, opening up the possibility of achieving high-speed 3D imaging simply

by scanning through a range of focal lengths. The drawback of this approach is that images of non-native planes suffer degraded image quality due to aberrations. Tuning the focal length of the tube lens corrects for the lowest order (defocus) aberration, but cannot correct for higher order aberrations (spherical, astigmatism, coma, etc). This is not due to imperfections in the tube lens: if the tube lens in isolation was fully aberration-corrected at each focal length in its range then the performance of the system would still be limited by the objective lens because it is not tuneable. Thus regardless of which tube lens is used, an objective lens that is not tuneable can only form an aberration-corrected image of a single object plane, as established by the Abbe sine condition.

Before reviewing the sine condition and exploring its implications for a non-focal imaging system we note that if the tube lens could be tuned to correct not just defocus but all the aberrations of the combined tube lens/objective system, then one could indeed achieve diffraction-limited volume imaging. However such a system presents an extreme engineering challenge, especially considering the NA values and fields of view for modern microscope objectives. Current tuneable lenses are far from meeting this requirement. Thus in the analysis below we quantify the severity of spherical aberration when imaging in non-native planes with a static objective lens and a tube lens that can be tuned to correct for system defocus. Since light is focused at infinity in the region between the two lenses, the distance between the lenses does not affect our analysis, and the system behaves as a single-lens system subject to the sine condition, diagrammed in the following page.

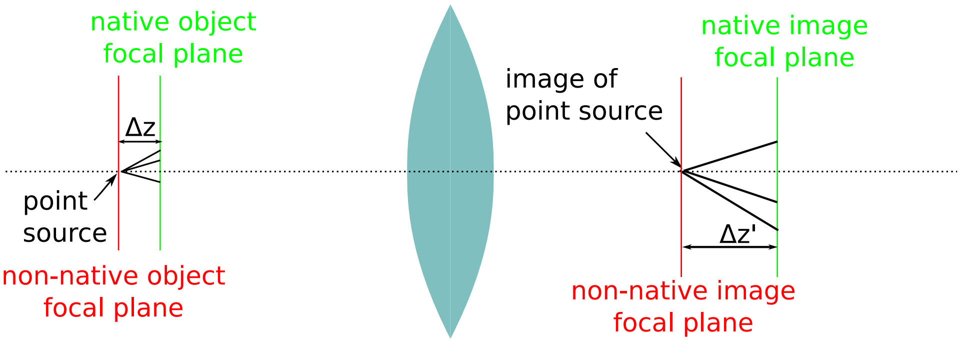

Consider a point source along the optic axis separated by Δz from the native object focal plane. Rays are emitted from this object over a range of angles and can be traced to the native image focal plane.

The sine condition states that in an aberration-corrected system each ray passing through the native object and image planes satisfies

$$n \sin\theta = M n' \sin\theta', \tag{1}$$

where n is the refractive index of the object immersion medium, n' that of the imaging immersion medium (here taken to be 1 for air), M the local linear magnification near the axis, and θ, θ' are the ray angles in the object and image space, respectively. For a ray of angle θ, let h denote the height of the strike position of each ray in the object focal plane, where $h = \Delta z \tan\theta$. We can calculate the position $\Delta z'_\theta$ where this intersects the optic axis:

$$\begin{aligned}
\Delta z'_\theta &= \frac{h'}{\tan\theta'} && (2)\\
&= \frac{Mh}{\frac{\sin\theta'}{\sqrt{1-\sin^2\theta'}}} && (3)\\
&= \frac{M\Delta z \tan\theta}{\frac{\frac{1}{M} n \sin\theta}{\sqrt{1-\frac{1}{M^2}n^2\sin^2\theta}}} && (4)\\
&= \frac{M^2}{n}\Delta z \frac{\sqrt{1-\frac{1}{M^2}n^2\sin^2\theta}}{\sqrt{1-\sin^2\theta}}. && (5)
\end{aligned}$$

Note that the strike position is a function of θ, and therefore in general we do not have perfect focus. The only time the angle-dependence disappears (and thus aberrations are avoided) is when the ratio of square-roots cancels, which requires $|M| = n$. This is the condition derived more generally by the Maxwell perfect-imaging theorem. (We can also see that the longitudinal magnification is $\frac{M^2}{n}$.)

When this condition does not hold, the fractional range of strike positions

(up to the maximum angle consistent with the numerical aperture NA) is

$$\frac{\sqrt{1-\frac{\mathrm{NA}^2}{M^2}}}{\sqrt{1-\frac{\mathrm{NA}^2}{n^2}}}-1 \tag{6}$$

For $M = 20$ and water immersion, at NA 0.5 we get approximately 8%, and at NA 1 we get a 50% spread.

The axial RMS spot radius offers a more informative summary of the imaging performance. This quantity is the square root of the mean squared error (MSE) in strike position for every ray within the collection cone of the objective lens. For a ray with angle θ emitted from a source at Δz the squared error is

$$(\Delta z'_\theta - z'_0)^2 = \left(\Delta z'_\theta - \frac{M^2}{n}\Delta z\right)^2 \tag{7}$$

For each angle θ an ideal point source at Δz emits a circle of rays. It can easily be shown with trigonometry that the total light collected at angle θ is proportional to $\tan(\theta)$. Therefore we scale the error at each angle θ accordingly to get the sum squared error and then divide to calculate MSE:

$$\mathrm{MSE} = \frac{\int_0^{\theta'} \tan(\theta)\left(\Delta z'_\theta - \frac{M^2}{n}\Delta z\right)^2 \mathrm{d}\theta}{\int_0^{\theta'} \tan(\theta)\,\mathrm{d}\theta} \tag{8}$$

$$= \frac{\int_0^{\theta'} \tan(\theta)\left(\Delta z'_\theta - \frac{M^2}{n}\Delta z\right)^2 \mathrm{d}\theta}{-\ln(\cos(\theta'))} \tag{9}$$

The upper limit, θ' is the maximum angle that the objective can collect as specified by the NA. Note that the above calculates the RMS spot radius in image space; in order to convert this to object space units divide by the axial magnification.

Supplementary Note 2: Microscope parts

(ordered approximately from excitation to collection of emission)

Number in diagram	Part description	Vendor	Custom?	Part number	Price (USD)	Count	
not shown	Laser system w/5 wavelengths, AOTF	Spectral (now Andor)	no	Laser Merge Module (LMM)	90000	1	*discontinued, estimated price
1	Pigtailed fiber collimator	OZ Optics	no	LPC-01-405/640-3/125-P-1-6AC-40-3S-3-2	146.5	1	
2	Pigtailed fiber collimator holder	(machine shop)	yes		NA	1	
3	Lightsheet lens module (“cigarette”)	(machine shop)	yes		NA		
not shown	Lightsheet achromat lens	Edmund Optics	no	45-262	87.5	1	
not shown	Lightsheet cylinder lens	Tower Optical	yes*		99	1	
not shown	Lightsheet achromat lens (thin sheet)	Edmund Optics	no	45-207	59	1	
not shown	Lightsheet cylinder lens (thin sheet)	Edmund Optics	yes**	48-373	375	1	
4	Rotation collar	(machine shop)			NA	1	
5	Clamp for lightsheet lens holder	(machine shop)			NA	1	
not shown	10x, 0.3 N.A. objective (UMPLFLN10X/W)	Olympus	no	1-U2M583	774.25	1	
6	20x, 0.5 N.A. objective (UMPLFLN20X/W)	Olympus	no	1-U2M585	1464.64	1	
not shown	40x, 0.8 N.A. objective (LUMPLFLN 40X/W)	Olympus	no		2500	1	
7	Objective holder (RMS)	(machine shop)	yes		NA		
8	Mini-dovetail stage (2-axis)	Lightspeed Technologies Inc.	no	MDE266	304	1	
9	Mini-dovetail stage (3-axis)	Lightspeed Technologies Inc.	no	MDE269	573	1	
10	dovetail stabilizer	(machine shop)	yes		NA	1	
11	Front piezo plate	(machine shop)	yes		NA	1	
12	Piezo positioner, 800um range	Piezosystem Jena	no	NanoSX800	11205	1	
not shown	Piezo amplifier (digital control)	Piezosystem Jena	no	30DV300	5616	1	
13	Rear piezo plate	(machine shop)	yes		NA		
not shown	Precision broadband mirror	Edmund Optics	no	48-017	395	1	
not shown	200mm tube lens	Thorlabs	no	ITL200	450	1	
not shown	Knife edged mirror	Thorlabs	no	MRAK25-G01	125.46	1	
not shown	50/50 beamsplitter (25 x 36 mm)	Thorlabs	no	BSW10R	110	1	
not shown	Filter cube w/insert	Thorlabs	no	DFM	304	1	
not shown	Extra filter cube insert	Thorlabs	no	DFMT1	201	1	
not shown	0.9x telecentric relay lens	Edmund Optics	no	62-902	2265	2	
not shown	CMOS camera	PCO	no	Edge 4.2	16400	2	
not shown	Stages for aligning cameras	Thorlabs	no	DTS25	179.5	4	
not shown	DAQ board (PCI-6259)	National instruments	no	779072-01	1592.1	1	
not shown	Physiology stage surface	Thorlabs	no	PHYS24BB	2500	1	
not shown	Lab jack	Newport	no	281	999.94	1	
not shown	Breadboard for connecting lab jack	Thorlabs	no	MB1224	259	1	
not shown	XY microscope translation stage	Scientifica	no	N/A (Quote ref: QLS-30894)	4042.5	1	
not shown	RAID hard drives	Seagate	no	4221403	78.19	20	
not shown	Breadboard for vertical mounting of system	Thorlabs	no	MB1824	400	1	
				Price total:	164194.69		

***Cylindrical lens specifications**

Diameter	3mm +0/-0.2mm
Center Thickness	1mm ±0.1mm
Edge Thickness	1.37mm
Effective Focal Length	-6.25mm
Back Focal Length	-6.91mm
Focal Length Tol	±3%
Radius	-3.24mm
Surface quality	60/40 both sides
Coating	MgF2

****Cylindrical lens specifications (thin sheet)**

Same as Edmund Optics 48-372, only diameter was reduced to 5mm

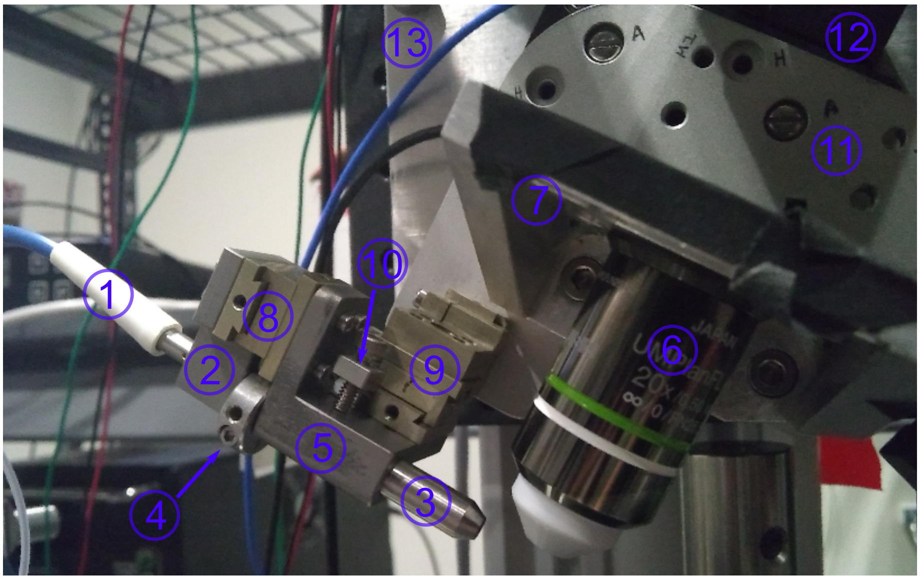

view from front

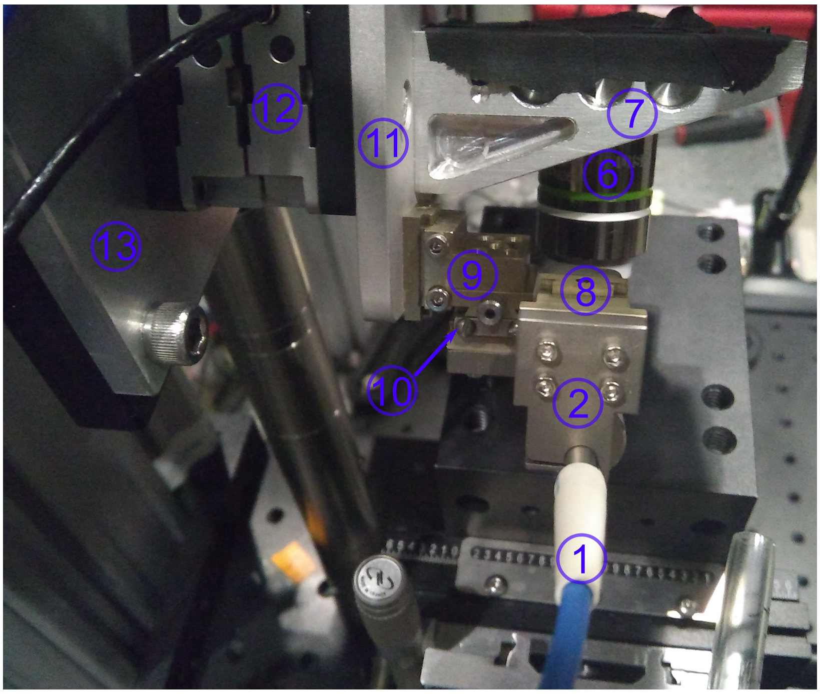

view from left

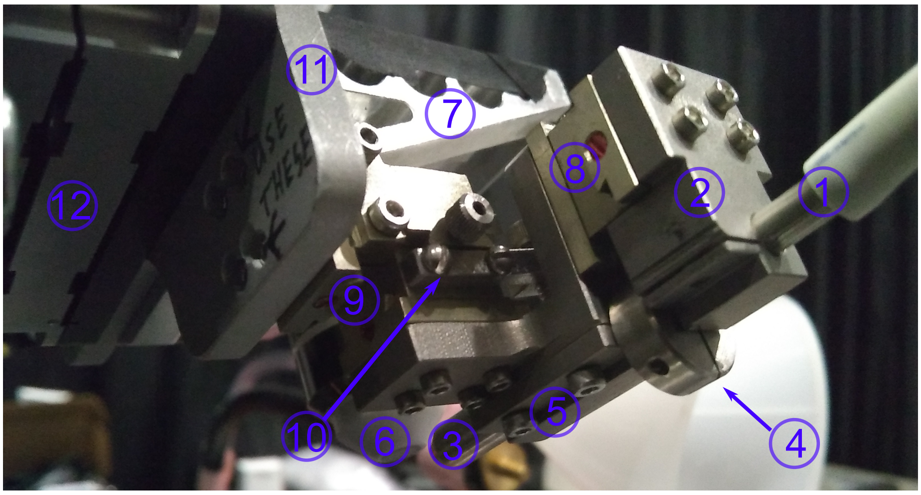

view from rear left

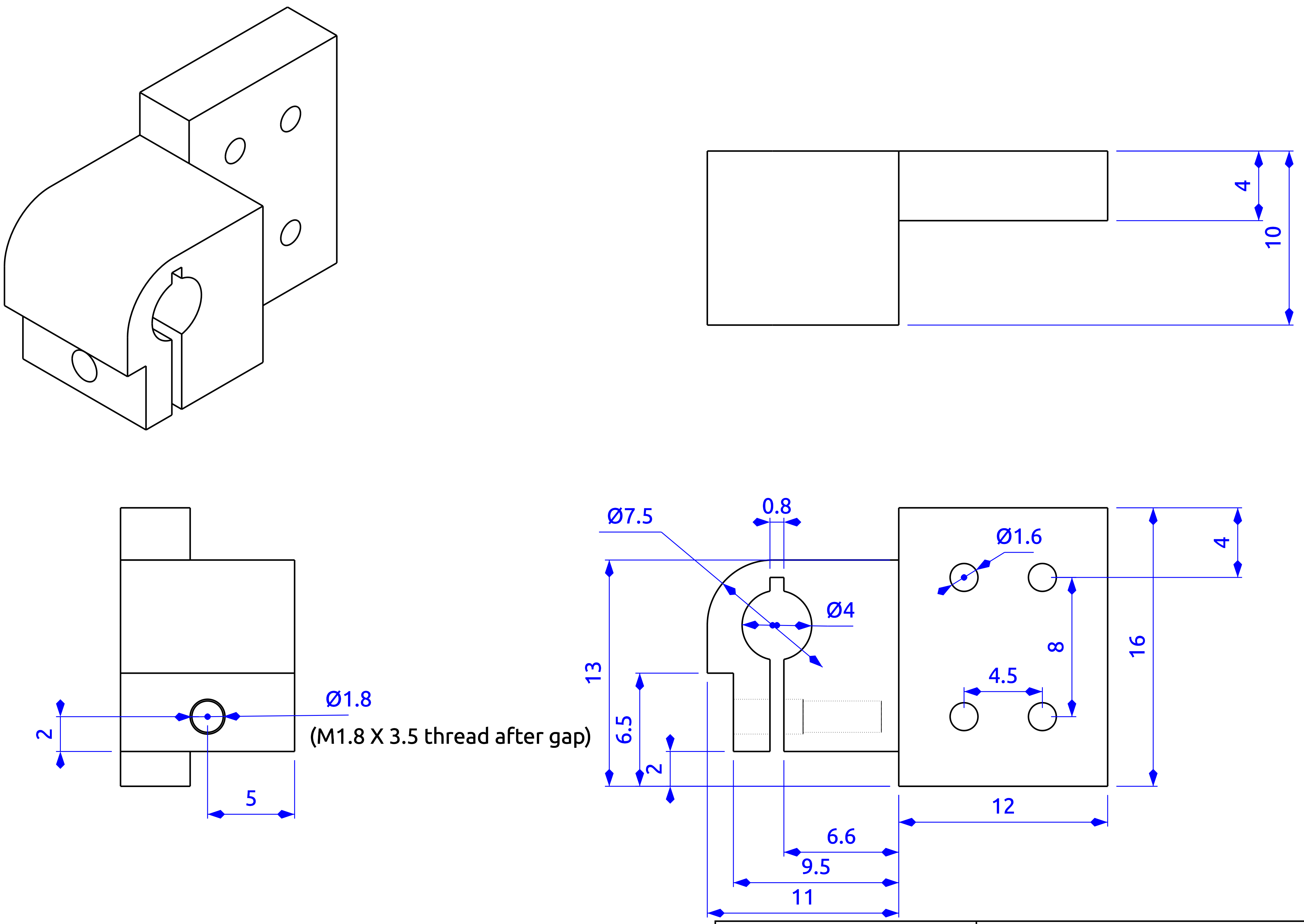

Created by: Cody Greer	Title: Pigtailed collimator holder		
Supplementary information: Clamps onto pigtailed collimator<br>Attaches to MDE266 dovetail slide<br>Addtl authors: T.E. Holy J. Kreitler K. Poenicke	Size: A3	Sheet: X / Y	Scale: mm
	Part number: NA		
	Drawing number: DN		
	Date: 17/10/2017	Revision: REV A	

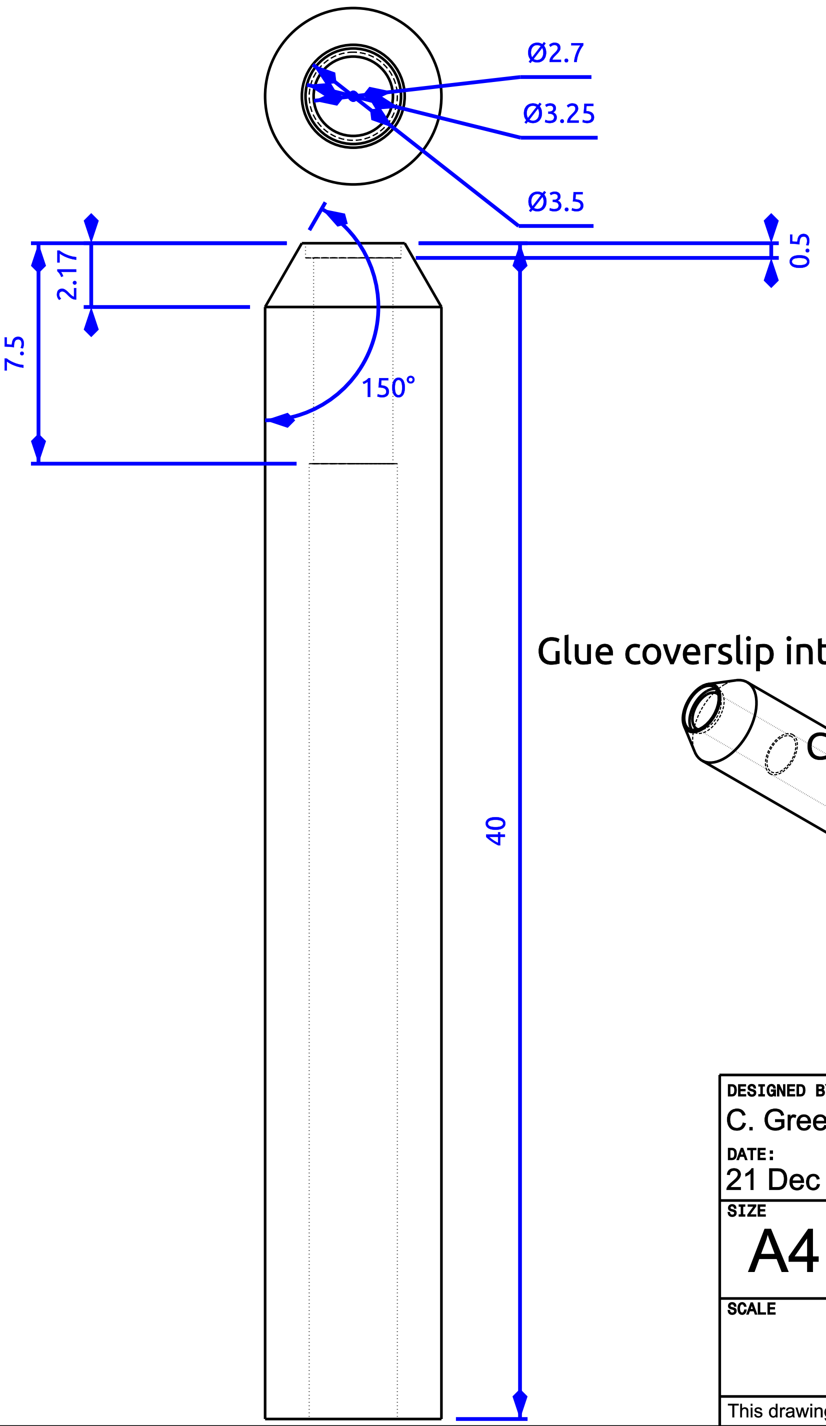

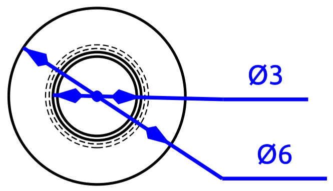

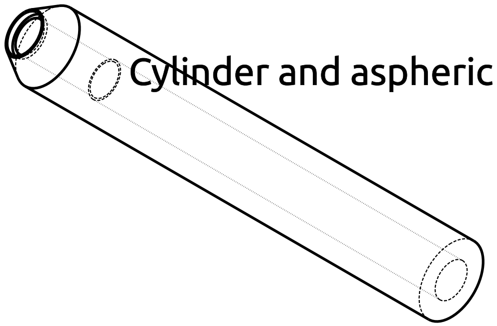

DESIGNED BY: C. Greer, T.E. Holy<br>DATE: 21 Dec 2017		Lightsheet Lens Module<br>"cigarette"	
SIZE A4			
SCALE	WEIGHT (kg)	DRAWING NUMBER<br>Addtl designers: J. Kreitler, K. Poenicke	SHEET

G	_
F	_
E	_
D	_
C	_
B	_
A	_

This drawing is our property; it can't be reproduced or communicated without our written consent.

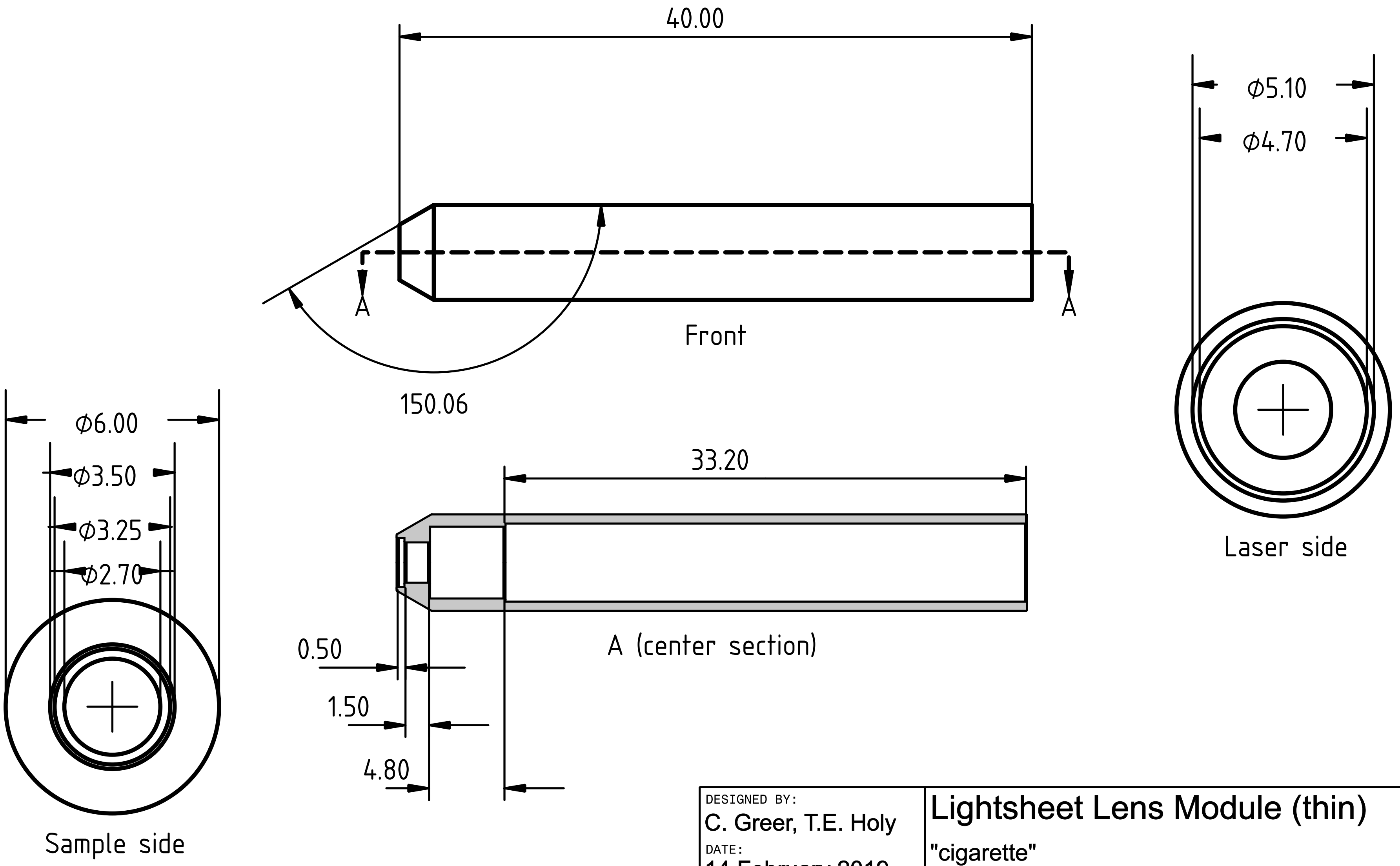

DESIGNED BY: C. Greer, T.E. Holy<br>DATE: 14 February 2019	Lightsheet Lens Module (thin)<br>"cigarette"	
SIZE A4		
SCALE mm	WEIGHT (kg)	DRAWING NUMBER + designers: J. Kreitler, K. Poenicke — SHEET

This drawing is our property; it can't be reproduced or communicated without our written consent.

G	_
F	_
E	_
D	_
C	_
B	_
A	_

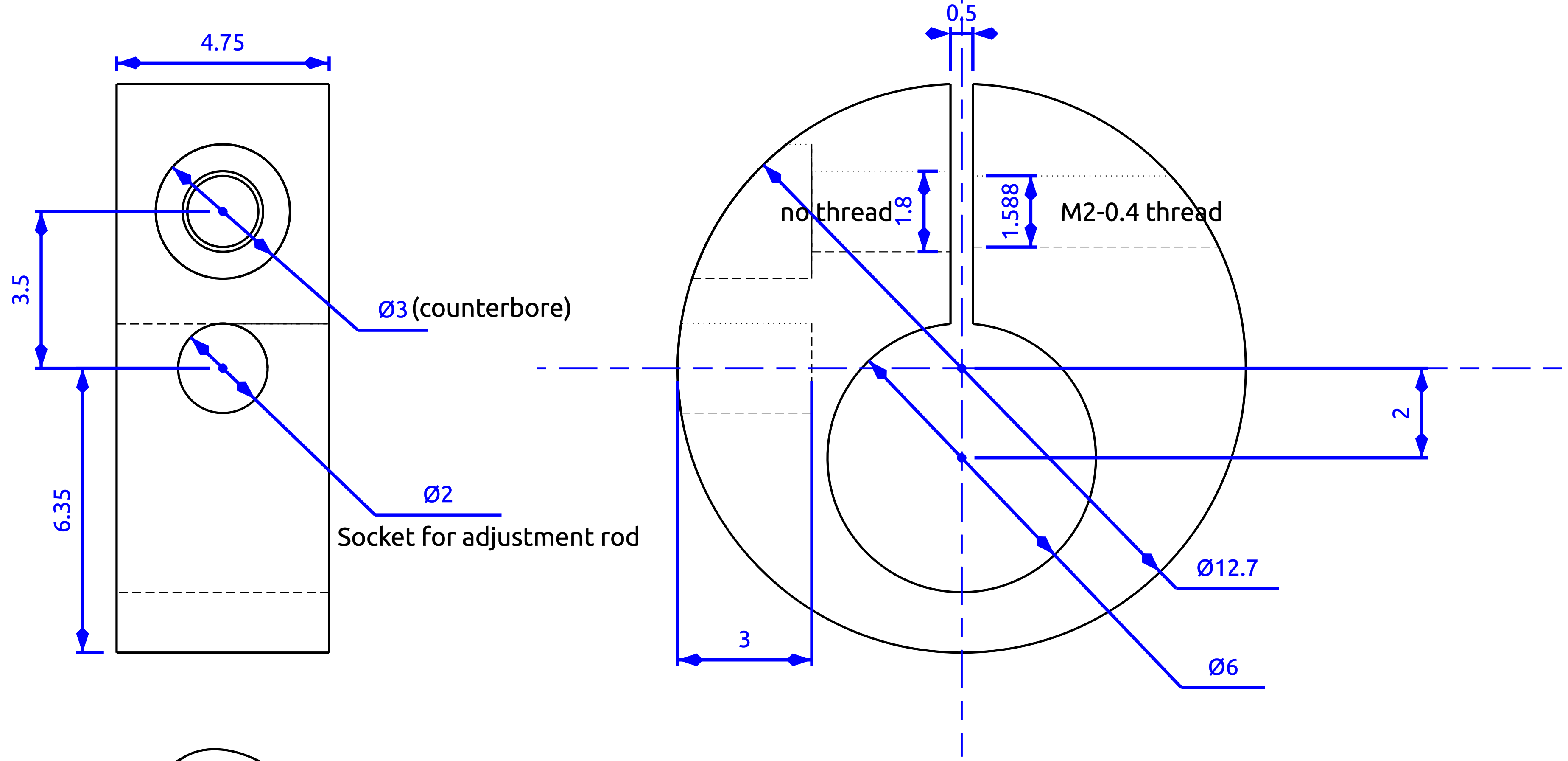

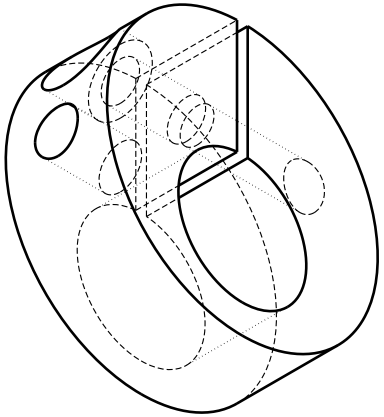

DESIGNED BY:
C. Greer, T. E. Holy

DATE:
22 December 2017

Rotation Collar

Attaches to lightsheet "cigarette"

SIZE
A4

SCALE

WEIGHT (kg)

DRAWING NUMBER
Addtl designers: J. Kreitler, K. Poenicke

SHEET

G	_
F	_
E	_
D	_
C	_
B	_
A	_

This drawing is our property; it can't be reproduced or communicated without our written consent.

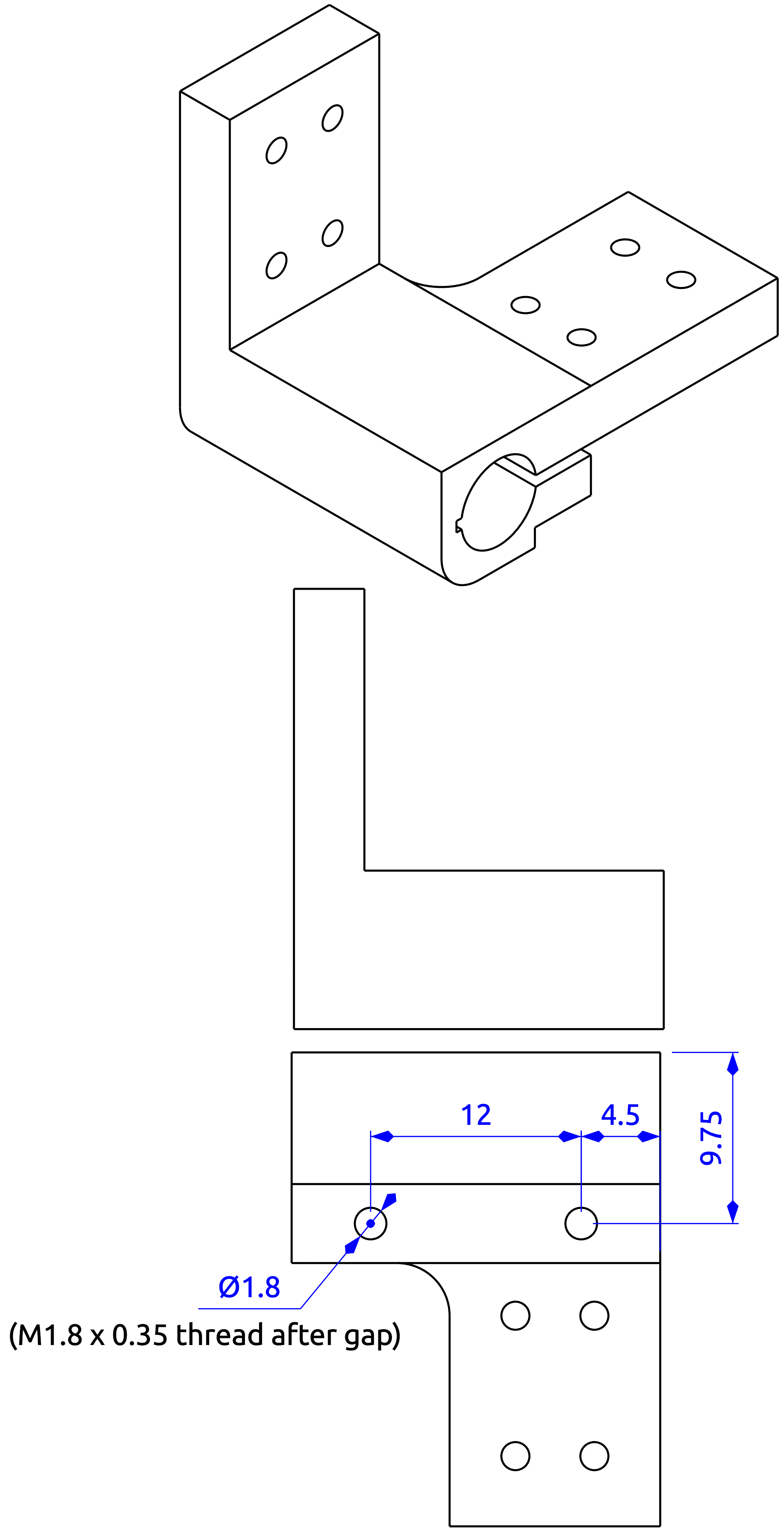

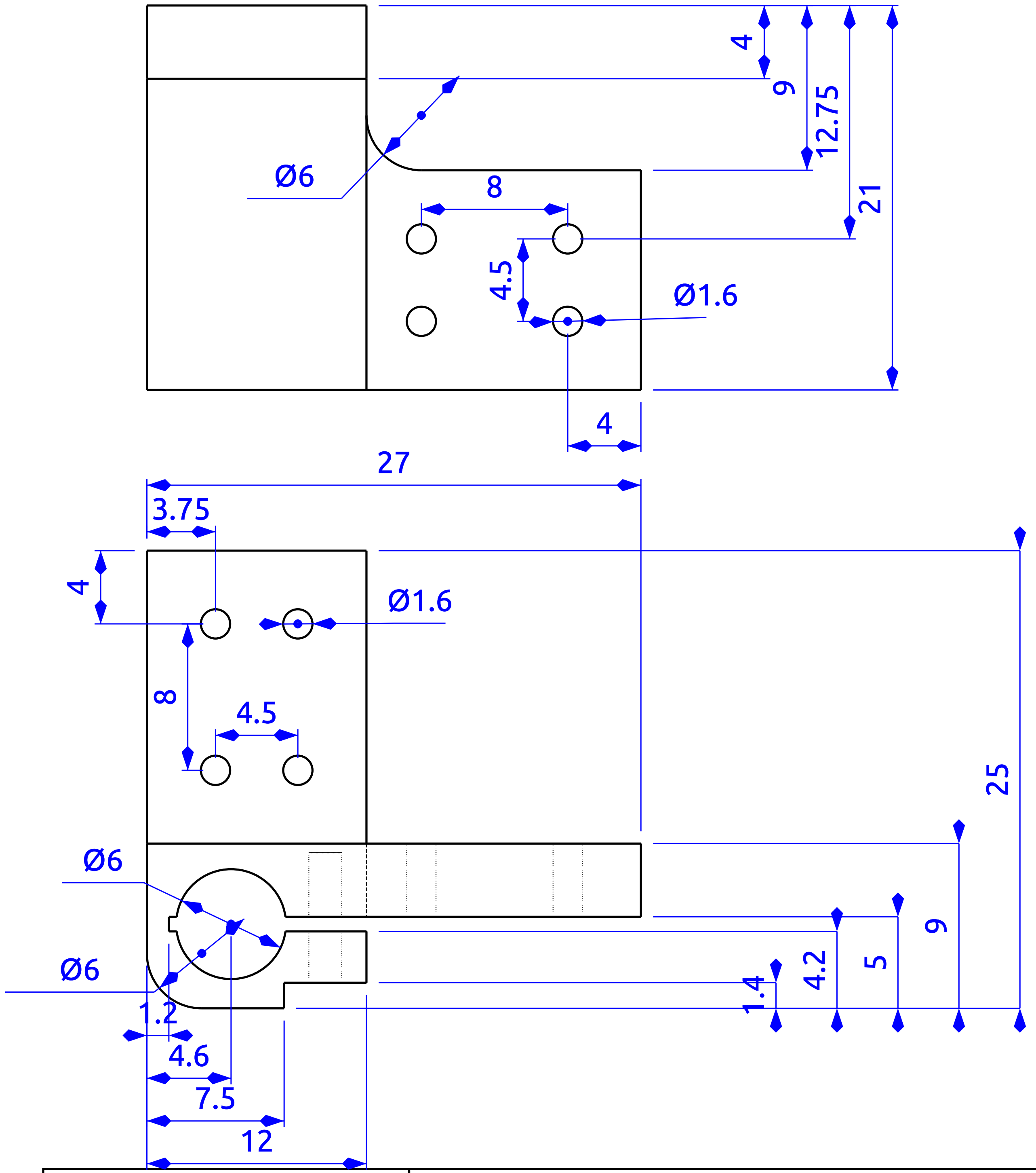

Created by: Cody Greer	Title: Clamp for light-sheet lens holder		
Supplementary information: Clamps around the light-sheet "cigarette" attaches to dovetail slides MDE266 MDE269 Addtl authors: T. E. Holy J. Kreitler K. Poenicke	Size: A3	Sheet: X / Y	Scale: mm
	Part number:		
	Drawing number: NA		
	Date: 18/10/2017		Revision: REV A

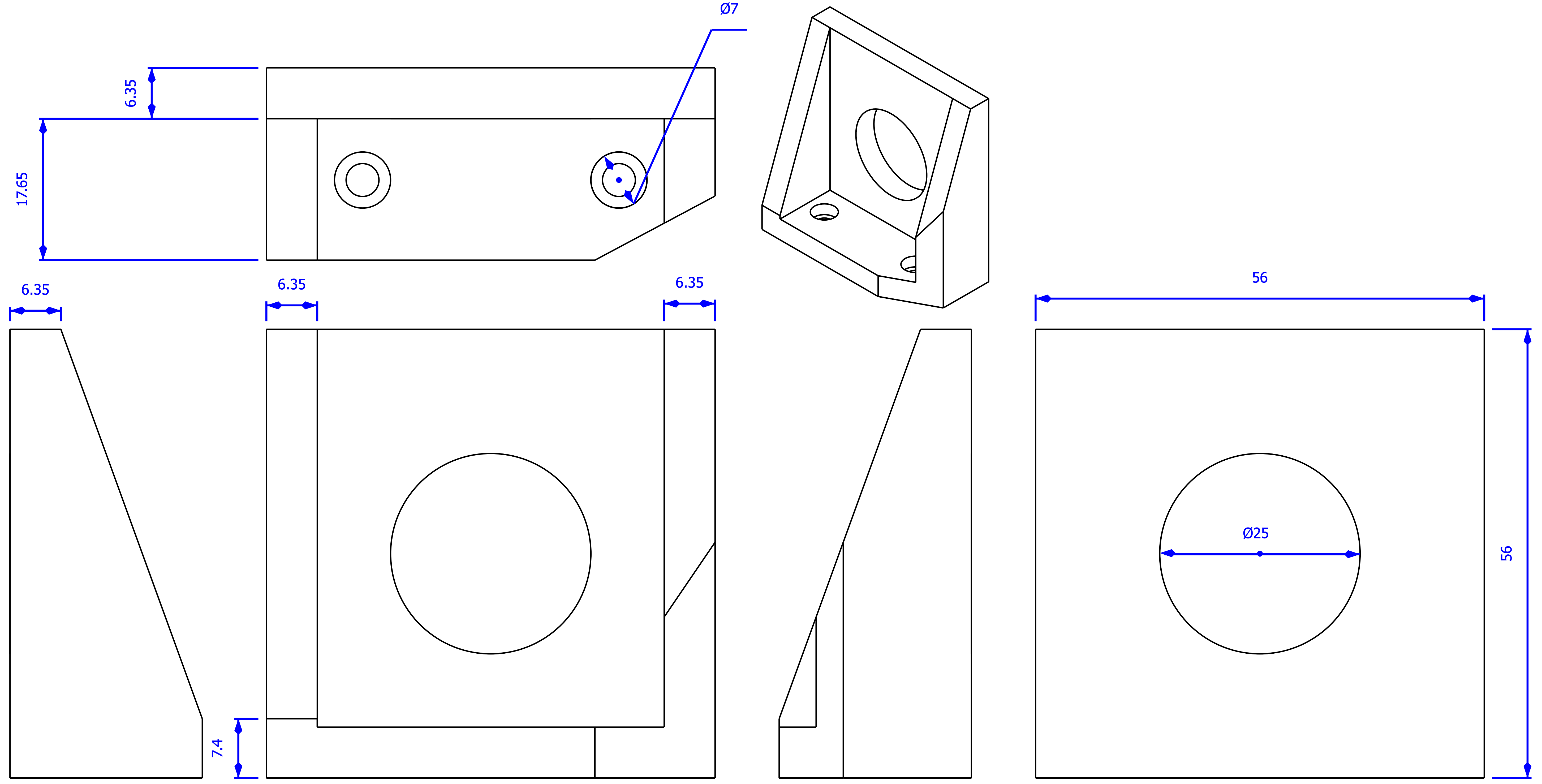

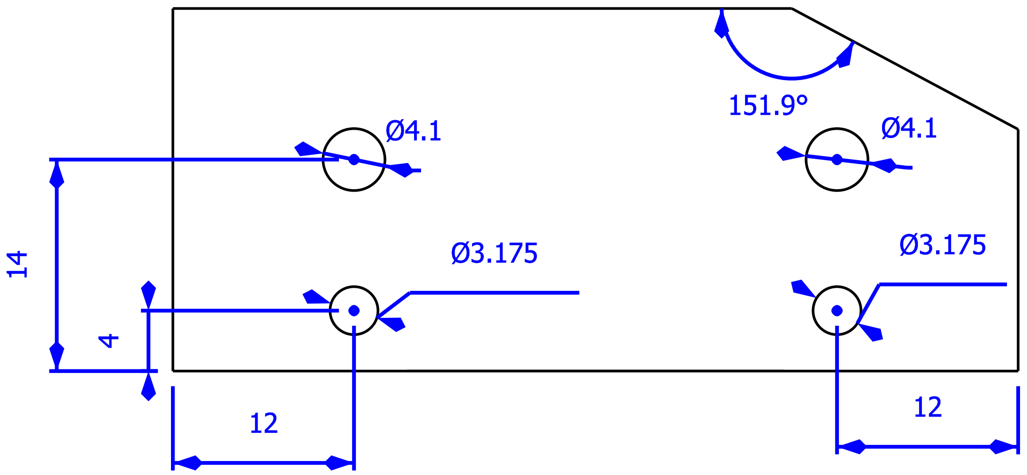

DESIGNED BY:
J Kreittler, TE Holy, C Greer
DATE:
2014
CHECKED BY:
SUPERVISOR NAME
DATE:
CHECK DATE

HOLDER FOR RMS OBJECTIVE

OCPI 2

SIZE			
A3			

SCALE	WEIGHT (kg)	DRAWING NUMBER	SHEET
SCALE	WEIGHT	NUMBER	SHEET

I	_
H	_
G	_
F	_
E	_
D	_
C	_
B	_
A	_

This drawing is our property; it can't be reproduced or communicated without our written agreement.

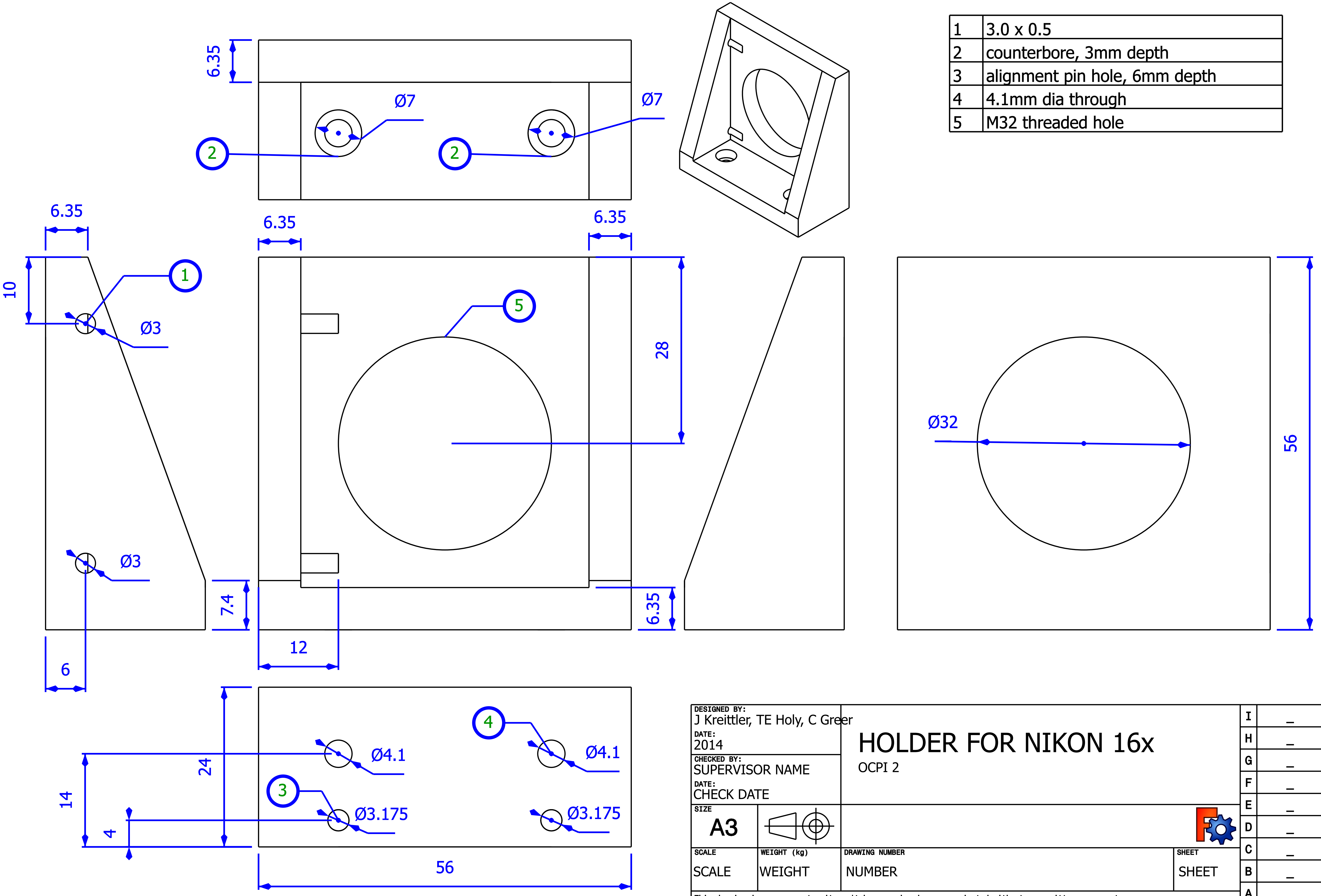

1	3.0 x 0.5
2	counterbore, 3mm depth
3	alignment pin hole, 6mm depth
4	4.1mm dia through
5	M32 threaded hole

HOLDER FOR NIKON 16x

OCPI 2

DESIGNED BY: J Kreittler, TE Holy, C Greer		
DATE: 2014		
CHECKED BY: SUPERVISOR NAME		
DATE: CHECK DATE		
SIZE A3		
SCALE: SCALE	WEIGHT (kg): WEIGHT	DRAWING NUMBER: NUMBER
SHEET: SHEET		

I	_
H	_
G	_
F	_
E	_
D	_
C	_
B	_
A	_

This drawing is our property; it can't be reproduced or communicated without our written agreement.

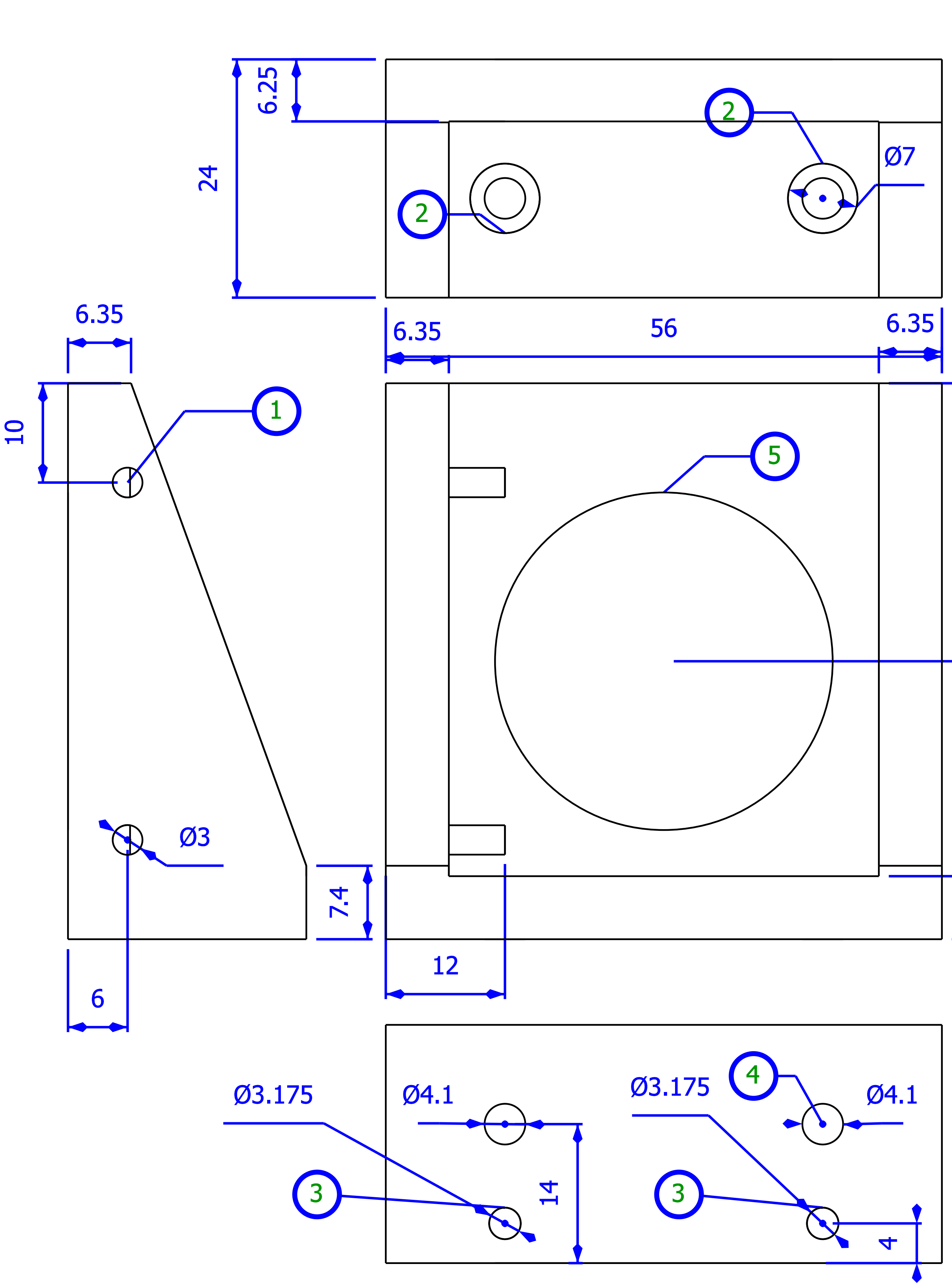

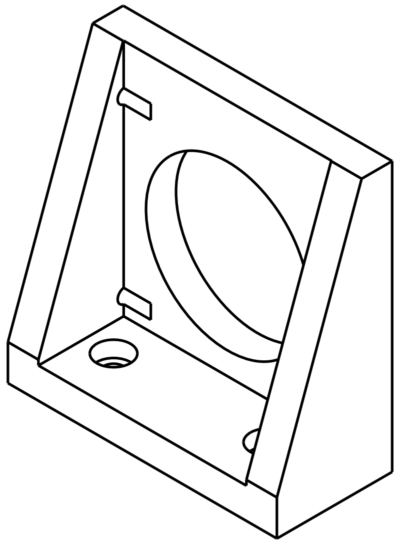

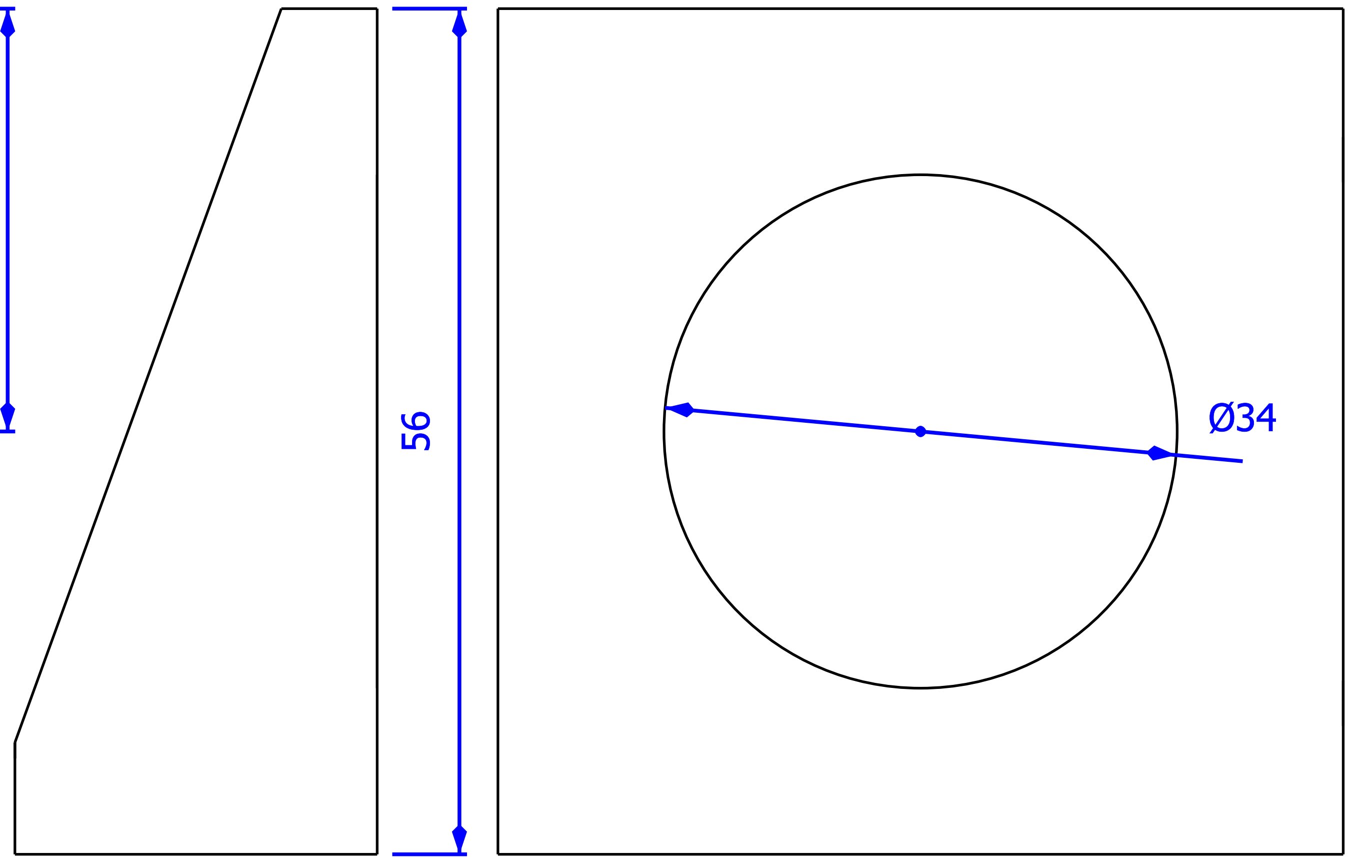

1	3.0 x 0.5
2	counterbore, 3mm depth
3	alignment pin hole, 6mm depth
4	4.1mm dia through
5	M34 threaded hole

DESIGNED BY:
J Kreittler, TE Holy, C Greer
DATE:
2014
CHECKED BY:
SUPERVISOR NAME
DATE:
CHECK DATE

HOLDER FOR OLYMPUS 10X MI

OCPI 2

SIZE
A3

SCALE
SCALE
WEIGHT (kg)
WEIGHT
DRAWING NUMBER
NUMBER
SHEET
SHEET

I	_
H	_
G	_
F	_
E	_
D	_
C	_
B	_
A	_

This drawing is our property; it can't be reproduced or communicated without our written agreement.

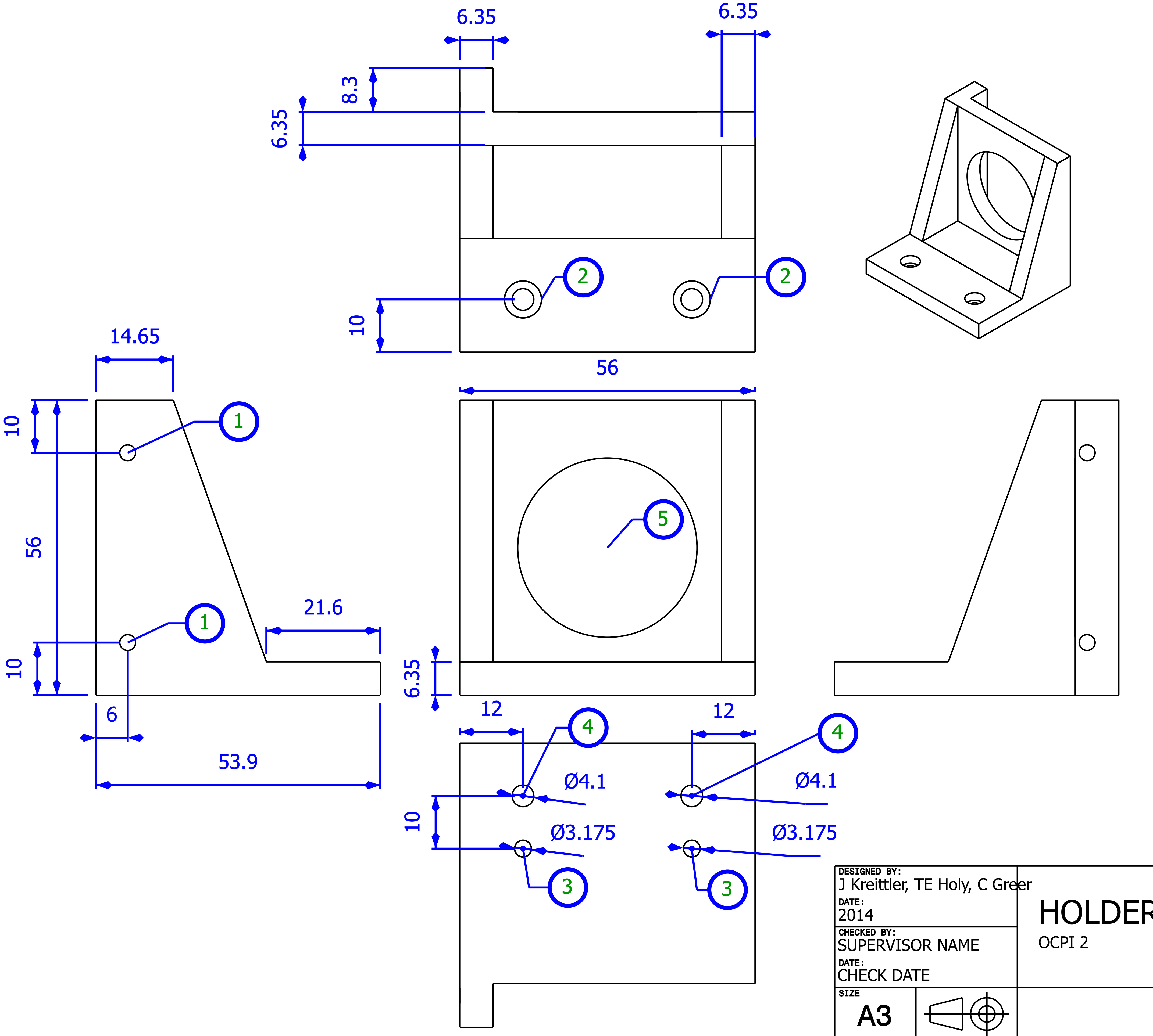

1	3.0 x 0.5 thread
2	7mm dia counterbore, 3mm depth
3	alignment pin hole, 6mm depth
4	4.1mm dia through
5	M34 threaded hole for objective

HOLDER FOR OLYMPUS 4x

OCPI 2

DESIGNED BY: J Kreittler, TE Holy, C Greer

DATE: 2014

CHECKED BY: SUPERVISOR NAME

DATE: CHECK DATE

SIZE: A3

SCALE: SCALE

WEIGHT (kg): WEIGHT

DRAWING NUMBER: NUMBER

SHEET: SHEET

I	_
H	_
G	_
F	_
E	_
D	_
C	_
B	_
A	_

This drawing is our property; it can't be reproduced or communicated without our written agreement.

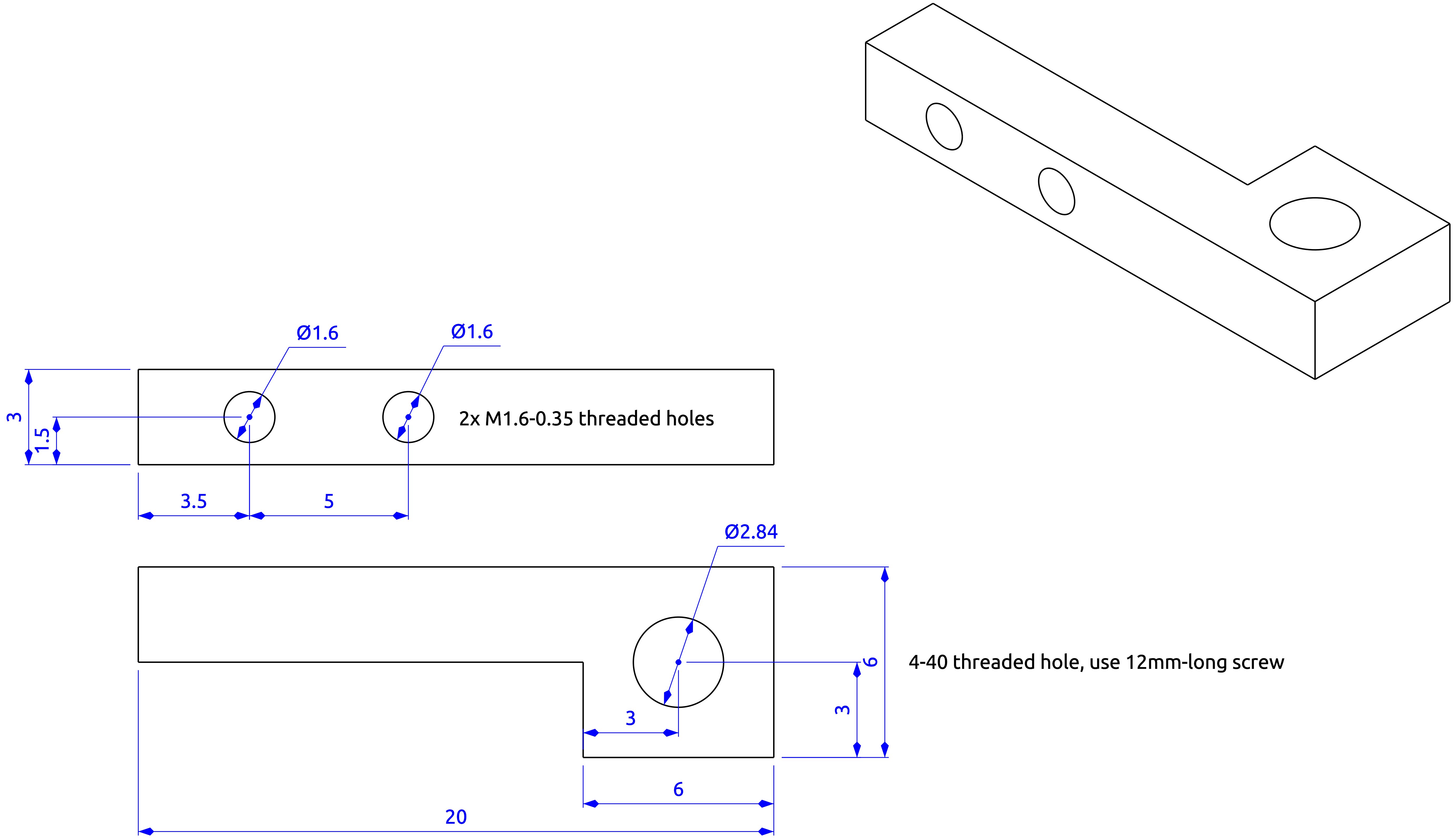

DESIGNED BY:
C. Greer, K. Poenicke
DATE:
1 November 2017
CHECKED BY:
NA
DATE:
NA

Dovetail stabilizer

Applies tension to dovetail joint

SIZE: A3

SCALE: mm

WEIGHT (kg): NA

DRAWING NUMBER: NA

SHEET: NA

This drawing is our property; it can't be reproduced or communicated without our written agreement.

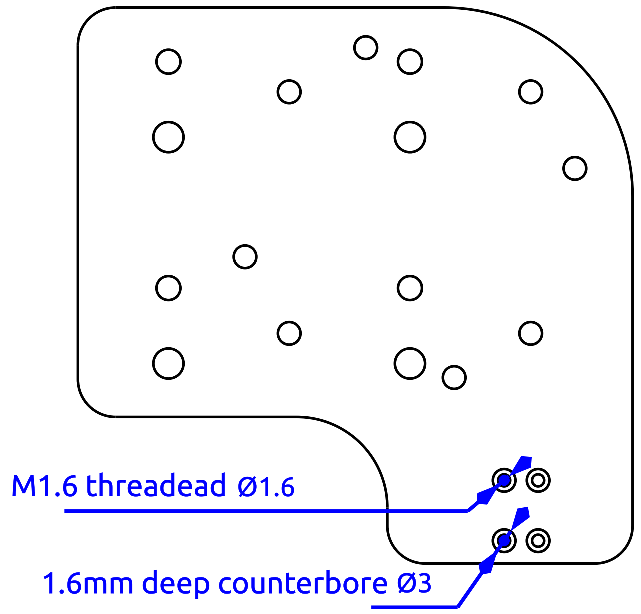

Rear view

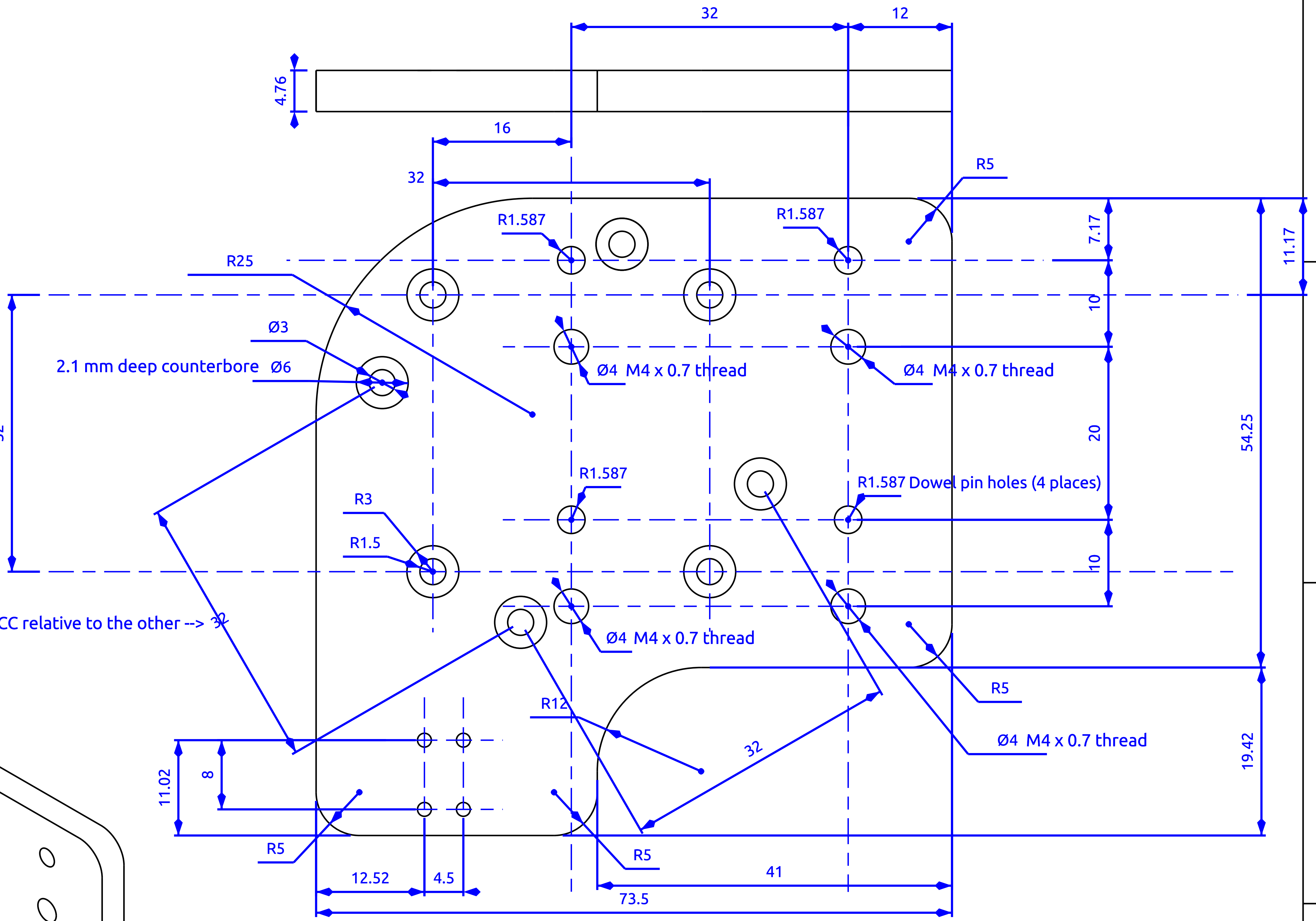

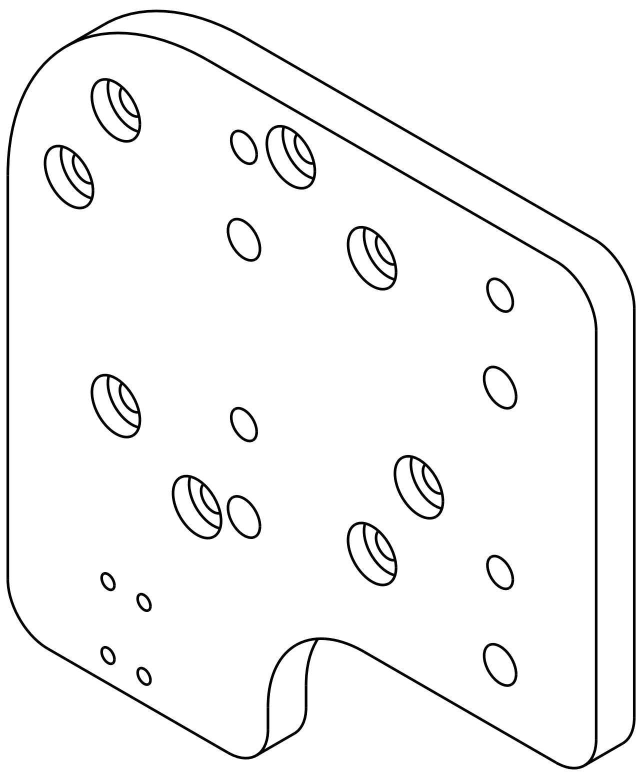

DESIGNED BY: Cody Greer, John Kreittler, Kevin Poenicke	**Front piezo plate** OCPI 2 microscope
DATE: 19 February 2017	
CHECKED BY: SUPERVISOR NAME	
DATE: CHECK DATE	
SIZE A3	
SCALE: SCALE	WEIGHT (kg): WEIGHT
DRAWING NUMBER: NUMBER	SHEET: SHEET

This drawing is our property; it can't be reproduced or communicated without our written agreement.

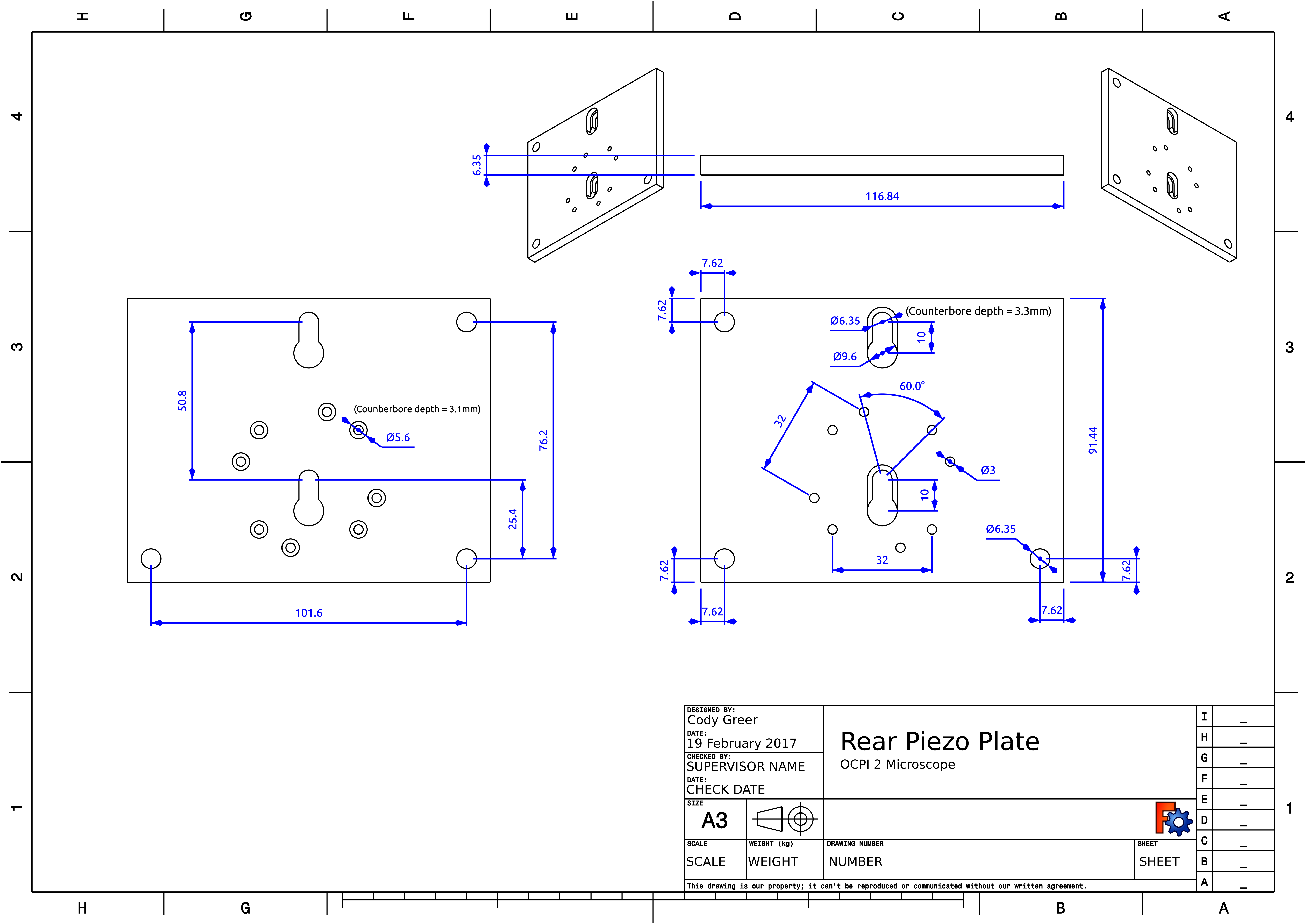

DESIGNED BY:
Cody Greer
DATE:
19 February 2017
CHECKED BY:
SUPERVISOR NAME
DATE:
CHECK DATE

Rear Piezo Plate

OCPI 2 Microscope

SIZE		DRAWING NUMBER	SHEET
A3			
SCALE	WEIGHT (kg)	DRAWING NUMBER	SHEET
SCALE	WEIGHT	NUMBER	SHEET

This drawing is our property; it can't be reproduced or communicated without our written agreement.

Supplementary Figure 1

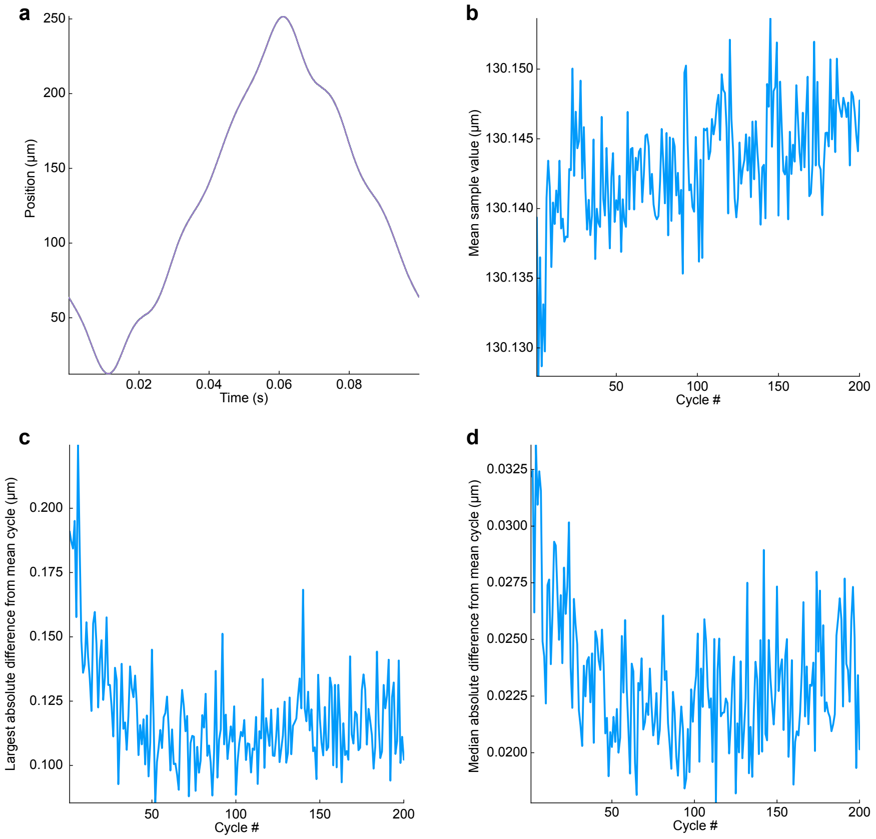

Supplementary Figure 1 Consistency of the piezo response **(a)** Overlay of 200 consecutive piezo scan cycles as measured by the capacitive sensor of the device (cycles during the first 20 s of operation are excluded). The command signal was a 10 Hz triangle wave. Samples were acquired at 100 kHz and downsampled to 10 kHz for plotting. **(b)** Closed-loop control of the piezo prevents drift ("creep") in the mean piezo response measured during each cycle from (a). **(c)** Piezo response cycles are also consistent on a per-sample basis after an initial settling period. First a mean response cycle was created by averaging corresponding samples across the 200 cycles. Plotted is the maximum absolute difference of any sampled value of each cycle from the corresponding value in the mean cycle. Before computing differences each cycle was lowpass filtered with a gaussian kernel of width 100 μs to reduce sampling noise. **(d)** Similar to (c), but the median difference between corresponding samples in each cycle is shown.

Supplementary Figure 2

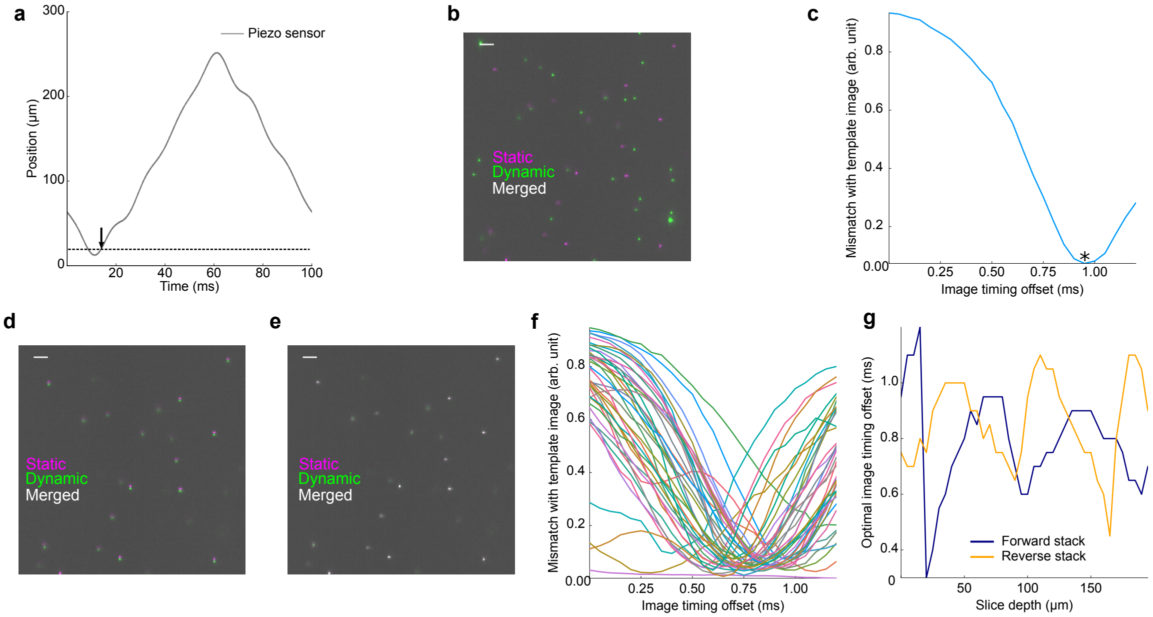

Supplementary Figure 2 Slice timing calibration **(a)** A single piezo response cycle as measured by the piezo's built-in capacitive sensor. The dashed line marks a position at which the user desires to acquire and image. An arrow marks the intersection of the line with the sensor trace. The timing of this intersection sets the initial guess for the timing of image acquisition. The procedure illustrated in panels b-e is applied independently for each such plane that the user desires to image. **(b)** The initial guess at camera timing based on the piezo sensor is inaccurate for fast dynamic recordings. Two images of the same bead sample were acquired: one during dynamic operation with the timing guess described in (a), and a second image, which serves as ground truth, was acquired at the same location during a very slow scan. The overlay of the two images shows poor correspondence. Scalebar: 5 µm. **(c)** The initial timing guess is refined by acquiring dynamic images at various temporal offsets and choosing the offset that yields an image matching the static template. The timing offset that yielded an image with minimal dissimilarity (see methods) is marked with an asterisk. **(d)** When the images corresponding to the optimal timing from (c) are overlaid they show good axial alignment, but the dynamic image exhibits a lateral shift. **(e)** A lateral translation is sufficient to align the dynamic image from (d) with the template. This lateral transformation is recorded and applied to align each corresponding slice of each stack in a timeseries recording. **(f)** When the same procedure is applied to align each 5 µm-spaced slice in a stack, slices vary in their optimal timing offset. **(g)** Optimal timing offsets are shown for each slice in both the forward and reverse stacks of a bidirectional recording. Note that optimal timings cannot be predicted by the depth in the sample, as would be expected from a simple error in sensor gain.

Supplementary Figure 3

a

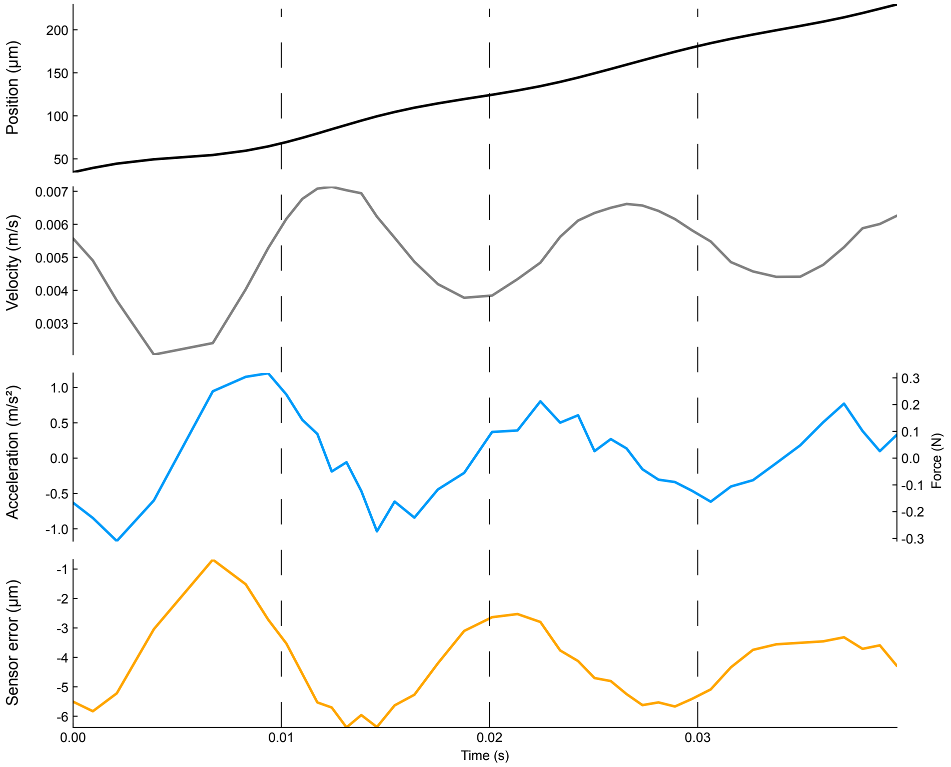

b

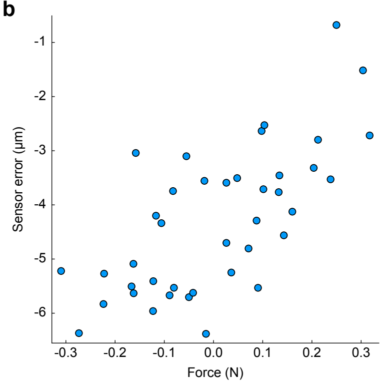

Supplementary Figure 3 Kinetic analysis of scan system **(a)** Error in the scanning position measurement is better predicted by the acceleration (or equivalently, force) of the scan system than by lower-order kinetics. Plotted are measured kinetic parameters of the piezo during the “forward” sweep of the cycle shown in Supplementary Figure 2a. Only the subset of timepoints corresponding with the timing of each image is plotted, and the raw piezo sensor trace was first lowpass filtered with a gaussian kernel of width 300 µs to reduce sampling noise. From top to bottom: position, velocity, acceleration. The third trace also shows estimated force based only on acceleration and the 264 g load of the piezo. The fourth and final trace shows for each image slice the difference between the measured position and the actual position of the focal plane as determined with the camera in the procedure described in Supplementary Figure 2. Note that this trace aligns better with the acceleration/force trace than with the other traces. **(b)** Scatter plot of points from the blue force trace in (a) showing that force is predictive of axial displacement of the focal plane.

Supplementary Figure 4

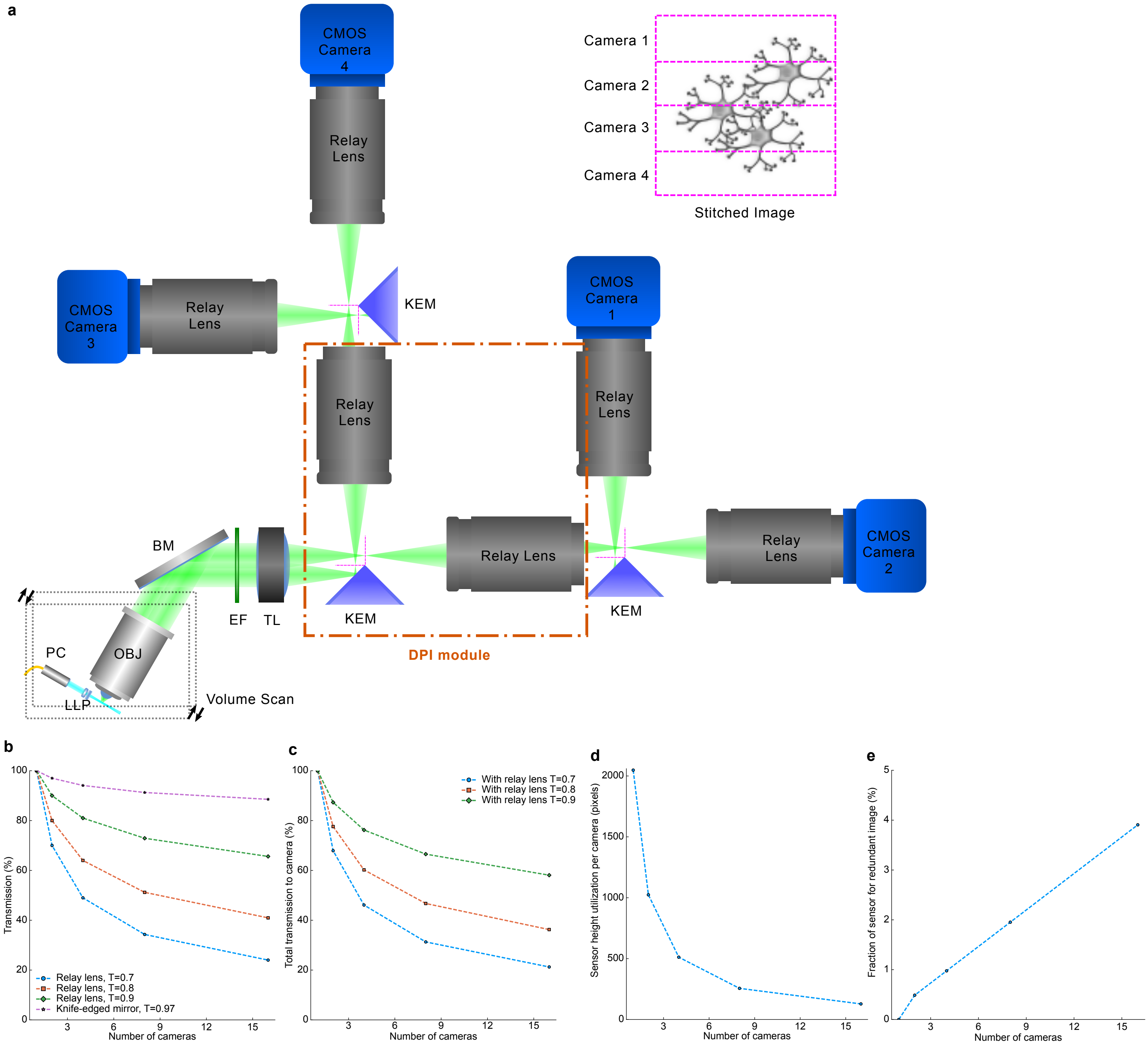

Supplementary Figure 4 Scalability of DPI **(a)** Multiple DPI modules can be chained together to split the image to more than two cameras. Shown is a diagram of an OCPI system with DPI scaled to four cameras. The fundamental repeating unit of the design is outlined in orange. Abbreviations are the same as in Figures 1a and 3a. **(b)** Each DPI module compounds losses to imperfections in the transmission and reflectivity of the relay lenses and the KEM, respectively. These losses are plotted separately as a function of the number of cameras in the system for relay lens transmission efficiencies ranging from 70% to 90%. **(c)** Same as (b) but KEM losses have been combined with transmission losses for each lens plotted. **(d)** As more cameras are added to the system less of the vertical extent of each sensor is utilized to acquire a full stitched image. As shown in Figure 1, the maximum framerate is inversely proportional the utilized vertical extent of the sensor. **(e)** The fraction of each image that contains redundant image information increases linearly with the number of cameras in the system. Thus the effective size of the field of view is reduced by a small amount when compared with a single-camera system.

Supplementary Figure 5

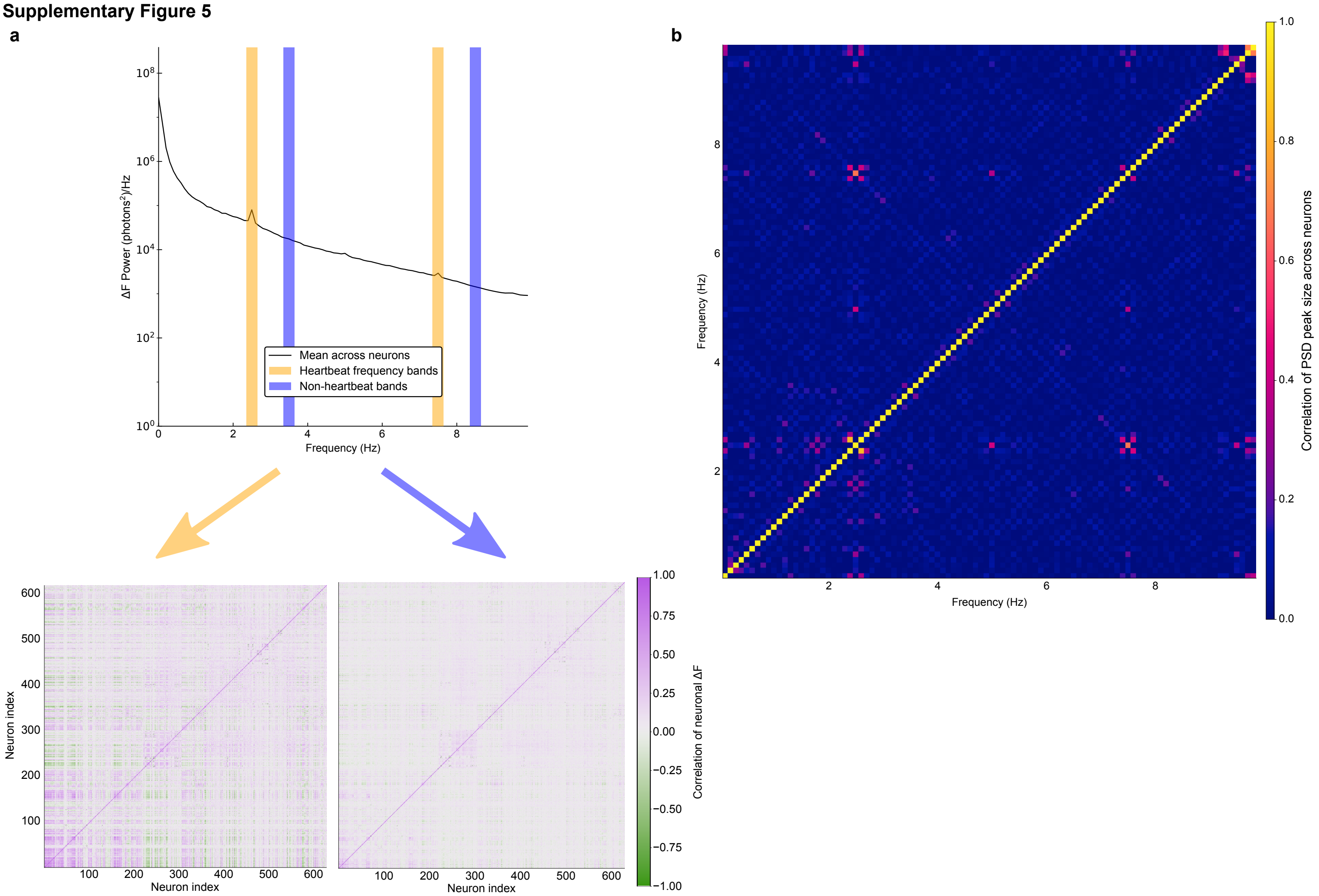

Supplementary Figure 5 Heartbeat-mediated correlations in the $\triangle$F signal **(a)** Comparison of time-domain correlations in the isolated heartbeat frequency bands (left) and another nearby set of frequency bands. The former correlation matrix contains a much higher proportion of strong positive and negative correlations, as would be expected with a broadly distributed heartbeat-induced motion signal. **(b)** The sizes of spectral deviations at the frequencies of the putative heartbeat signal are highly correlated across neurons, suggesting that the two frequencies are components of a single signal. For each of the 629 segmented neurons we measured the size of the peak in the PSD at each frequency. Peak amplitiude was measured by dividing the PSD amplitude at each frequency by the mean amplitude of the two surrounding frequency bins (thus peak size is not defined for the maximum and minimum frequency bins). Plotted is the correlation between peak sizes across frequencies. Correlations near the diagonal are expected to be strong because they are between similar frequencies. However the strong correlation between the 7.5 Hz and 2.5 Hz peak size stands out. Correlation is also visible at 5.0 Hz (another harmonic of 2.5 Hz).

Supplementary Figure 6

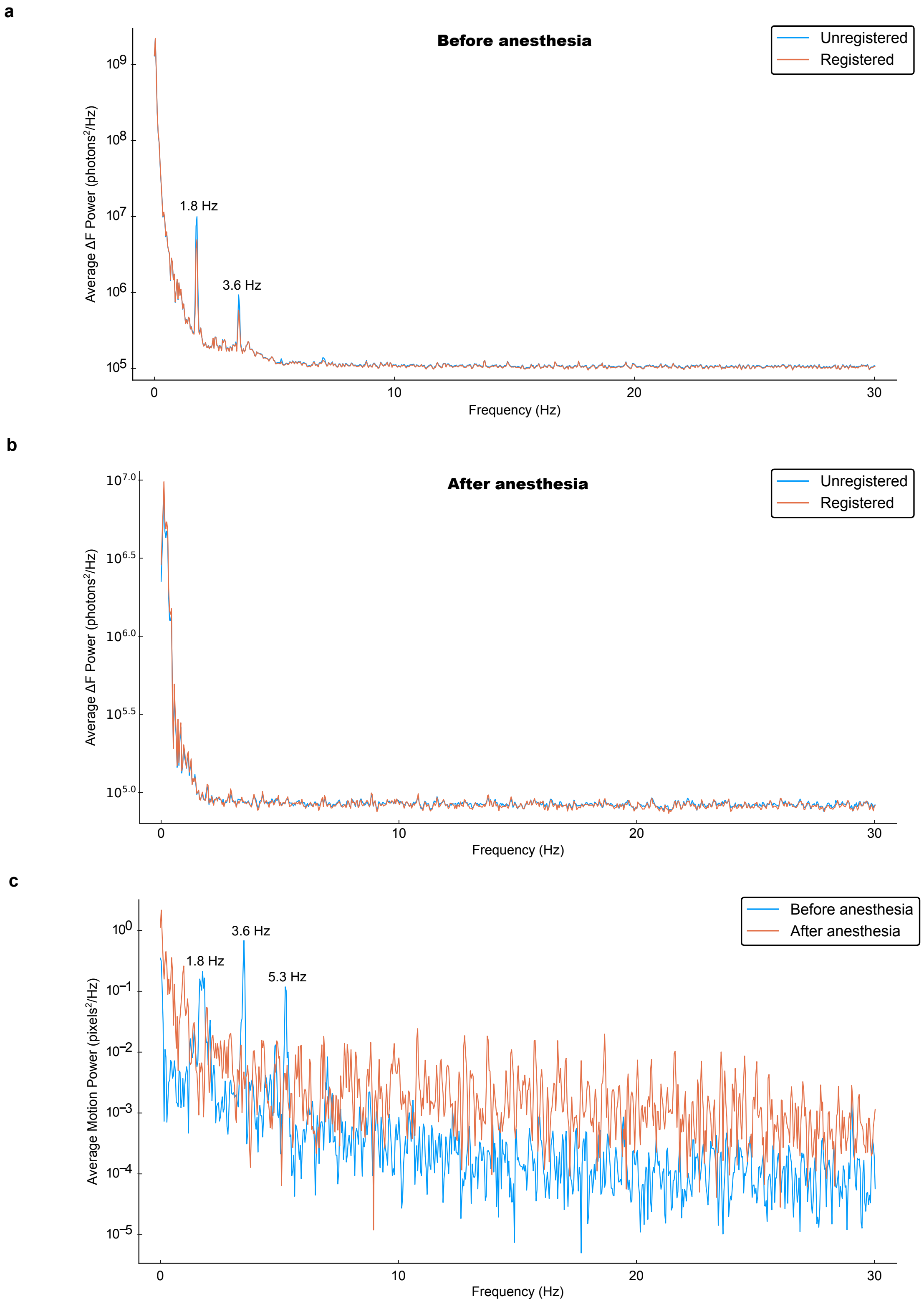

Supplementary Figure 6 Changes in power spectra due to anesthetic and image registration **(a)** Spectral power averaged across image ROIs during single-plane imaging of the larval zebrafish forebrain at 100 Hz (This is a different individual than the fish shown in Supplementary Figure 5). Putative heartbeat-related peaks are visible at 1.8 Hz and the 3.6 Hz harmonic (see methods for details of ROI selection). The difference in fundamental frequency (1.8 Hz) when compared to 2.5 Hz for the fish from Supplementary Figure 5 can be explained by individual variability in heart rate. Image registration diminishes the magnitude of the peaks but does not remove them. **(b)** The fish from (a) was imaged again after administering a heart-stopping dose of anesthetic (tricaine). The putative heartbeat-related peaks from (a) are fully attenuated, confirming that the signal originates from physiology rather than microscope hardware. **(c)** A power spectral analysis of the changing magnitudes of pixel translation vectors over time (translations found by registering video frames from (a) and (b)). Peaks in the pre-anesthetic power spectrum correspond with the image intensity peaks from (a), confirming that the artifact originates primarily from specimen motion. Note that peaks have magnitude less than 1, indicating that the artifact results from sub-pixel motion.
