# Supplementary material for: Fast objective coupled planar illumination microscopy
Source: Nat Commun. 2019 Oct 2;10:4483. doi: 10.1038/s41467-019-12340-0 (PMC6775063; doi:10.1038/s41467-019-12340-0)
Supplement: Supplementary file 3 — Description of Additional Supplementary Files [file 41467_2019_12340_MOESM3_ESM.pdf]

Description of Additional Supplementary Files

File Name: Supplementary Movie 1
Description: Whole-brain scans of a 5dpf HuC:GCaMP6s larval zebrafish are rendered plane-by-plane in the order recorded by the microscope. Green and magenta pseudocolor distinguishes the two halves of the stitched image acquired in a 2-camera DPI configuration. The recording was made at 10 Hz volume rate; playback of the video has been slowed 10×. The size of the imaged volume was 1020 µm × 348 µm × 200 µm, with a 0.65 µm × 0.65 µm × 5 µm voxel size.

File Name: Supplementary Movie 2
Description: Neural activity within individual slices of a 40-slice volume encompassing the forebrain of a 5dpf HuC:GCaMP6f larval zebrafish is visualized in realtime. A volume of size 223 µm × 127 µm × 200 µm was imaged at 20 Hz volume rate and 0.65 µm × 0.65 µm × 5 µm voxel size. ΔF values are displayed in magenta, baseline fluo- rescence is in grayscale. This video is an excerpt from the 20-minute analyzed recording.
